# Greenhouse gas emissions intensity of food production systems and its determinants

**DOI:** 10.1371/journal.pone.0250995

**Published:** 2021-04-30

**Authors:** Aldona Mrówczyńska-Kamińska, Bartłomiej Bajan, Krzysztof Piotr Pawłowski, Natalia Genstwa, Jagoda Zmyślona

**Affiliations:** Faculty of Economics, Department of Economics and Economic Policy in Agribusiness, Poznan University of Life Sciences, Poznań, Poland; Shenzhen University, CHINA

## Abstract

It is estimated that about 1/4th of all greenhouse gas (GHG) emissions may be caused by the global food system. Reducing the GHG emissions from food production is a major challenge in the context of the projected growth of the world’s population, which is increasing demand for food. In this context, the goal should be to achieve the lowest possible emission intensity of the food production system, understood as the amount of GHG emissions per unit of output. The study aimed to calculate the emission intensity of food production systems and to specify its determinants based on a panel regression model for 14 countries, which accounted for more than 65% of food production in the world between 2000 and 2014. In this article, emission intensity is defined as the amount of GHG emissions per value of global output. Research on the determinants of GHG emissions related to food production is well documented in the literature; however, there is a lack of research on the determinants of the emission intensity ratio for food production. Hence, the original contribution of this paper is the analysis of the determinants of GHG emissions intensity of food production systems. The study found the decreased of emission intensity from an average of more than 0.68 kg of CO_2_ equivalent per USD 1 worth of food production global output in 2000 to less than 0.46 in 2014. The determinants of emission intensity decrease included the yield of cereals, the use of nitrogen fertilizers, the agriculture material intensity, the Human Development Index, and the share of fossil fuel energy consumption in total energy use. The determinants of growth of emission intensity of food production systems included GDP per capita, population density, nitrogen fertilizer production, utilized agriculture area, share of animal production, and energy use per capita.

## Introduction

From an environmental point of view, particular significance can be ascribed to the reduction of greenhouse gas (GHG) emissions, which negatively impact all components of the environment and are responsible for climate change [1, 2]. An Intergovernmental Panel on Climate Change (IPCC) special report, *Global Warming of 1*.*5°C* [3], showed that anthropogenic GHG emissions have significantly contributed to climate change. All sectors of the economy are the source of these emissions. Particularly significant is the emission of pollutants generated by energy production and heating, industry, and the agri-food sector [4]. Agricultural production alone accounts for approximately 10–12% of all anthropogenic GHG emissions, and it is still increasing [5]. However, the share of total GHG emissions varies by continent. Europe is responsible for approximately 11% of the global GHG emissions from agriculture, Asia for approximately 44%, Africa for 15%, Australia and Oceania for 4%, and North and South America for 9% and 17%, respectively [6].

If GHG emissions from agriculture are increased by the amount generated by the rest of the food production sector, it is estimated that it may even account for about 1/4 of all anthropogenic GHG emissions [7]. Due to such a high carbon footprint, the environmental performance of food production should be an important element of climate change policy [8]. Pollution has a negative impact on human health and life and the environment; it also leads to higher economic costs [9]. For example, the use of excessive amounts of fertilizer results in pollution that harms the climate and the ecosystem [10] and, above all, causes soil degradation, which creates costs associated with lower agricultural productivity [11].

However, emissions in food production systems vary between continents and countries [12]. This is largely due to the directions of production, i.e., the differentiation between the unit emissions from animal production and plant production [13] and the general level of agricultural production intensity. As indicated by numerous studies [14–17], it is possible to reduce pollutant emissions through the phenomenon of *dematerialization*, i.e., by reducing the share of natural resources in the production of goods and increasing the share of information, i.e., research and technological progress. Examples of these activities include the use of energy from unconventional sources, increasing energy efficiency, or changes in the productivity and material intensity in the food production chain [18].

It is crucial that the fight against climate change does not hinder the growth of food production [19]. This is particularly important in light of the projected growth of the world’s population of about two billion by 2050 and another billion by 2100 [20]. Therefore, the overarching goal is to reduce the GHG emissions associated with food production while increasing food production [21]. To date, the research on simultaneously reducing emissions and increasing food at a level that meets the projected population growth (for example, by increasing yields) has reported mixed results [22]. In turn, the increase in the area of organic farming, which has been advocated by some circles and which reduces GHG emissions mainly by reducing the use of synthetic fertilizers, would not provide enough food with existing technology [23].

The conclusion of the research on the level of GHG emissions and food production is that the lowest possible emission rate per unit of output should be pursued [24]. In the literature, this indicator is called emission intensity or carbon intensity; it is related to the concept of carbon footprint, which in its general form assumes that the carbon footprint is a certain amount of GHG emissions that are considered to be related to an activity, for example, production [25]. Thus, the lower the emission intensity of the food production system, the fewer emissions per unit of production; by reversing this rate, it is possible to determine the environmental performance of food production.

So far, the research on emission intensity has mainly focused on comparing the performance of different cultivation systems in the case of plant production [26–28] or breeding systems in the case of animal production [29–31]. Few studies have considered the economic aspects in terms of emissions per production value [32]. The main conclusion of the research on the environmental performance of food production is the significantly higher emission intensity from animal production in comparison to plant production [33]. Animal production has a low environmental performance mainly due to high emissions at the breeding stage, as well as a higher degree of processing at a later stage of production [34].

Energy intensity is also a good approximation of the emission intensity of a food production system [35]. Based on research using this indicator, Pelletier et al. [36] noted that a significant source of GHG emissions in food production in the United States (US) includes emissions from energy consumption in the processing industry, including cooking, chilling, and freezing, which accounts for 15–20% of the total energy consumption in the US food system, also including distribution. Research on the determinants of GHG emissions associated with food production is well documented [37]; however, there is a lack of research on the determinants of emission intensity for food production. Specifying these determinants allows for verifying which factors have a stronger impact on the production in comparison to the increase in GHG emissions, which is important from the point of view of the possibility of decreasing the emissions per production unit.

The present study aimed to calculate the emission intensity of food production systems and specify its determinants. Emission intensity is understood as the emission of the main GHGs associated with food production divided by the value of this production. Fourteen countries ranked in the top 20 in terms of agricultural production in the world were examined. According to data from the Food and Agriculture Organization of the United Nations (FAO), these 14 countries account for over 65% of the world’s food production. The availability of uniform input-output tables limited the choice of countries; therefore, those with significant agricultural production for which such tables were available in the World Input-Output Database (WIOD) were selected. These included Australia, Brazil, Canada, China, France, Germany, India, Indonesia, Italy, Mexico, Russia, Spain, Turkey, and the US. This selection is intended to enable an analysis of countries that are important in terms of food production in the world market.

The rest of this article is organized as follows: Section 2 presents the data and research methods; Section 3 presents the research results and their discussion; and Section 4 presents a summary of the analysis.

## Methodology and data

### Data sources and carbon footprint

Two coherent data sources were used in the study: the WIOD, Release 2016 [38], where national input-output tables are available for 2000–2014, and WIOD Environmental Accounts Update 2000–2016 [39], which stores sectoral data on pollutant emissions. The above data sources are fully compatible with each other and they provide methodologically unified accounts classified according to the International Standard Industrial Classification (ISIC), Revision 4 (ISIC Rev. 4) [40]. Comprehensive information on the WIOD’s structure is provided by Dietzenbacher et al. [41], Timmer et al. [38], and Timmer et al. [42].

First, the carbon dioxide (CO_2_) emissions from the food production system for each country were calculated, following the Bajan and Mrówczyńska-Kamińska [43] method, which is based on Input-Output Life Cycle Assessment methodology. This method was used to measure emissions related to production; therefore, the boundary is set at the food industry activity level. Food production system CO_2_ emissions were grouped based on their origin, as follows:

- provision (materials used in the food industry and agriculture from other sectors);
- agriculture;
- food industry.

Based on ISIC Rev. 4, agriculture is represented by Sector A01: Crop and animal production, hunting, and related service activities. In turn, the food industry was defined as Sectors C10–C12: Manufacture of food products, beverages, and tobacco products. To determine CO_2_ emissions from the food production system, the carbon footprint coefficient was determined, as follows:

$$CCF_{i}=F_{CO_{2}i}/O_{i}$$ (1)

where:

*CCF*_*i*_ = carbon footprint coefficient of sector *i*

*E*_*CO2i*_ = CO_2_ emissions of sector *i*

*O*_*i*_ = output of sector *i*

The obtained coefficients were multiplied by the value of the inputs supplied by the sectors corresponding to agriculture and the food industry, as retrieved from the input-output tables. The values of the food production CO_2_ emissions were calculated using the following formula:

$$FPE_{CO_{2}}=\sum_{i=1}^{n}(z_{ia}*CCF_{i})+\sum_{i=1}^{n}(z_{if}*CCF_{i})-(z_{afa}*CCF_{a})-(z_{faf}*CCF_{f})+AE_{CO_{2}}+FIE_{CO_{2}}$$ (2)

where:

*FPE*_*CO2*_ *=* CO_2_ emissions from food production

*z*_*ia*_ = inputs from sector *i* to agriculture

*z*_*if*_ = inputs from sector *i* to the food industry

*z*_*afa*_ = inputs from agriculture to the food industry and agriculture

*z*_*faf*_ = inputs from the food industry to agriculture and the food industry

*CCF*_*a*_ = agriculture carbon footprint coefficient

*CCF*_*f*_ = the food industry carbon footprint coefficient

*AE*_*CO2*_ = agriculture emissions of CO_2_

*FIE*_*CO2*_ = the food industry emissions of CO_2_

Subsequently, methane (CH_4_) and nitrous oxide (N_2_O) emissions from agriculture were added to the CO_2_ emissions. To make the data comparable, the emission values were expressed in CO_2_ equivalents. Relevant data came from the FAO database [44]. The following conversion rates were applied: 21 for CH_4_ and 310 for N_2_O based on the Global Warming Potential of the IPCC Second Assessment Report [44]. The CH_4_ and N_2_O emission sources included enteric fermentation, manure management, rice cultivation, synthetic fertilizers use, manure applied to soils, manure left on pastures, crop residues, cultivation of organic soils, burning of crop residues, and savanna burning. Thus, the food production carbon footprint is the sum of food production CO_2_ emissions and the amount of CH_4_ and N_2_O pollution originating from agriculture, as follows:

$$FPCF=FPE_{CO_{2}}+AE_{CH_{4}}+AE_{N_{2}O}$$ (3)

where:

*FPCF* = food production carbon footprint

*AE*_*CH4*_ = agriculture emissions of CH_4_

*AE*_*N2O*_ = agriculture emissions of N_2_O

### Emission intensity

To examine the emission intensity of food production, the global output of food production was calculated using the Mrówczyńska-Kamińska and Bajan [45] method. This method assumes that food production consists the agriculture activities, the food industry activities, and other sectors’ activities that deliver inputs and services to agriculture and the food industry. Thus, calculated output refers to a system consisted of food manufacturing and food processing, starting with production of the necessary inputs. WIOD’s national input-output tables were used for the calculations. The output of food production was calculated as the total output of: aggregate I (provision, which is the value of materials used in the food industry and agriculture from other sectors), aggregate II (agriculture output), and aggregate III (the food industry output).

To calculate global output of aggregate I, agriculture intermediate consumption and the food industry intermediate consumption were summarized. Self-supply values were subtracted to avoid double counting, as follows:

$$O_{I}=IC_{a}+IC_{f}-z_{aa}-z_{ff}$$ (4)

where:

*O*_*I*_ = output of aggregate I

*IC*_*a*_ = agriculture intermediate consumption

*IC*_*f*_ = the food industry intermediate consumption

*z*_*aa*_ = agriculture self-supply value

*z*_*ff*_ = the food industry self-supply value

The global output of aggregate II was calculated by subtracting the agriculture inputs value to the food industry (which is a component of the global output of aggregate I) from the global output of agriculture, namely:

$$O_{II}=O_{a}+z_{af}$$ (5)

where:

*O*_*II*_ = output of aggregate II

*O*_*a*_ = agriculture output

*z*_*af*_ = agriculture inputs to the food industry

Similarly, the global output of aggregate III was calculated by subtracting the food industry inputs value to the agriculture sector (which is a component of the global output of aggregate I) from the global output of the food industry, namely:

$$O_{III}=O_{f}+z_{fa}$$ (6)

where:

*O*_*III*_ = output of aggregate III

*O*_*f*_ = the food industry output

*z*_*fa*_ = the food industry inputs to agriculture

Then, the emission intensity of food production was calculated as the ratio between the food production carbon footprint and the global output of food production as follows:

$$EIFP=FPCF/O_{FP}$$ (7)

where:

*EIFP* = emission intensity of food production

### Panel regression

The analysis of the influence of the selected factors on emission intensity was conducted based on the panel regression model. The research material was a macro panel containing data on 14 countries over a period of 15 years. The variables for the panel were selected based on the literature review; they are presented in Table 1.

**Table 1:** Variables used to estimate the panel regression model.

Variables	Description	Unit
GDP_per_capita	-	PPP, constant 2017 international $
HDI	Human Development Index	[0–1]
nitrogen_fertilizers_use	use in agriculture	tonnes/1000 ha of agricultural land
nitrogen_fertilizers_production	-	tonnes/1000 ha of agricultural land
agriculture_material_intensity	agriculture intermediate consumption/agriculture global output	%
food_industry_material_intensity	food industry intermediate consumption/food industry global output	%
energy_use_per_capita	energy used in an economy per capita	kilograms of oil equivalent per capita
fossil_fuel_energy_use	share of energy from fossil fuels used in an economy	%
population_density	-	people per sq. km of land area
agricultural_area	-	in 1000 ha
agricultural_area_per_person	-	ha/person
yield_of_cereals	-	tonnes/ha
animal_production	share of total agricultural production	%

Source: Author’s compilation.

Most often, panel regression models are either fixed-effects models [46]:

$$y_{it}=x_{it}\beta '+\alpha_{i}+\varepsilon_{it}$$ (8)

where:

*α*_*i*_(i = 1…. n) is the unknown intercept for each entity (n entity-specific intercepts),

*y*_*it*_ is the dependent variable, where *i* = entity and *t* = time, *x*_*it*_ is independent variable, *β*’ is the coefficient for the independent variable, and *ε*_*it*_ is the error term,

or random-effects models [47]:

$$y_{it}=\gamma +x_{it}\beta '+v_{i}$$ (9)

where:

*γ* is an absolute term,

*v*_*i*_ = *α*_*i*_+*ε*_*it*_ is the total random component, i.e., a random variable, which is the sum of an individual random component *α*_*i*_ and white noise *ε*_*it*_.

The assumption of the fixed-effects model is the occurrence of unknown (unobservable), but constant over time, differences between the units of the random-effects model. A specific random variable is assigned to each unit, the realization of which is responsible for the individual effect in a given period. In the random-effects model, individual effects are not the same in subsequent periods. Consequently, individual effects are not treated as parameters and their value is not estimated. While in the fixed-effects model, individual effects could be interpreted as an individual absolute term, different for each unit but constant over time, in the random-effects model, individual effects can be interpreted as individual random components.

The country data used in the study suggest that an estimation of the fixed-effects model should be carried out, as there are some differences between the countries that remain constant over time. A Hausman test [48] was conducted to confirm the validity of the assumption. The Hausman test allows for comparing two estimators:

*β*^1^ characterized by higher efficiency (lower variance) and
*β*^2^ characterized by lower efficiency.

The test’s null hypothesis postulates the compatibility of both estimators (variable-effects model), while the alternative hypothesis assumes the lack of compatibility of the estimator with higher efficiency and the estimator with lower efficiency (fixed-effects model). The formal statistics of the Hausman test are as follows:

$$H_{0}={\left(\beta^{2}-\beta^{1}\right)}^{T}{\left[Var(\beta^{2})-Var(\beta^{1})\right]}^{-1}(\beta^{2}-\beta^{1})$$ (10)

The test results indicated that the null hypothesis *(p-value = 0*.*000*) should be rejected in favor of an alternative hypothesis, indicating that a fixed-effects model should be used.

In the fixed-effects model, it is important to identify the problem of heteroscedasticity and autocorrelation. Heteroscedasticity occurs when the residual variance changes with the change of the independent variable *x*. This means that random disturbances are not equally distributed around the expected zero value. To identify heteroscedasticity, a modified Wald statistic [49] was used, the null hypothesis of which can be expressed as follows:

$$H_{0}:\sigma_{i}^{2}=\sigma^{2}\;for\;i=1,\ldots ,N$$ (11)

The test results indicated that the null hypothesis (*p-value = 0*.*000*) should be rejected, suggesting homoscedasticity in favor of the alternative hypothesis indicating heteroscedasticity.

Autocorrelation refers to a correlation between one variable with the same variable from another period or object. This is especially true for models estimated based on time-series data. When an autocorrelation of a random component occurs, the variance matrix is not diagonal because the random components for observations from different periods are correlated, not independent. To check if the autocorrelation is present in the model, a bias-corrected test for panel autocorrelation described by Born and Breitung [50] and Wursten [51] was performed. On this basis, the null hypothesis *(p-value = 0*.*000*) was rejected in favor of the alternative hypothesis, suggesting that there is an autocorrelation problem in the model.

Therefore, it was decided to develop a robust Panel Corrected Standard Errors (PSCE) model, which eliminates the issues of heteroscedasticity and autocorrelation [52, 53]. This model is suitable for a panel where the number of time-series periods is greater than the number of objects. The calculations were performed using STATA 15.

## Results and discussion

### Emission intensity results

**Fig 1:**
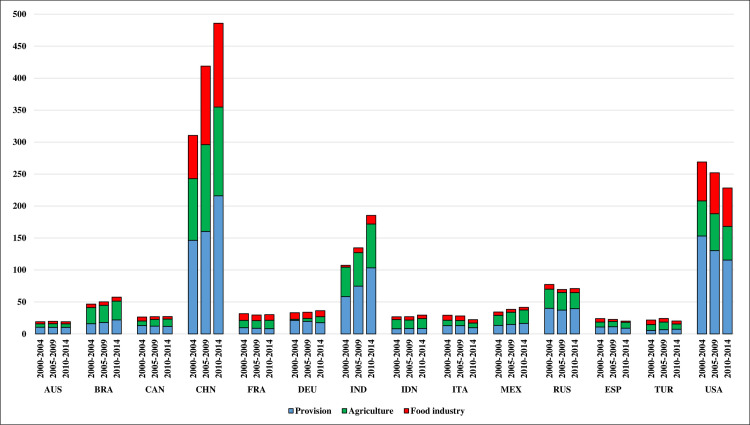
Average food production system carbon dioxide (CO_2_) emissions in megatonnes in the examined countries. Source: Authors’ calculations based on data from WIOD Environmental Accounts (Update 2000–2016) and WIOD, Release 2016.

The total CO_2_ emissions from food production in the 14 examined countries increased by more than 250 megatonnes (Mt = 10^6^ tonnes) from about 1 gigatonne (Gt = 10^9^ tonnes) in 2000 to almost 1.28 Gt in 2014. China is responsible for the largest increase in CO_2_ emissions (Fig 1). Detailed data from the input-output tables indicate that the main reason for the increase in emissions in China was the increase in intermediate consumption from the sectors producing energy, fertilizers, and pesticides. CO_2_ emissions from food processing and, to a lesser extent from agriculture, have also increased significantly. Similar to China, significant increases in emissions have also been recorded in India, mainly due to an increase in the intermediate consumption of energy, fertilizers, and pesticides. Moreover, among the examined countries, CO_2_ emissions also increased in Brazil, Canada, Germany, Indonesia, and Mexico. CO_2_ emissions from food production without any clear trend have been observed in Australia, France, Russia, and Turkey. In turn, Italy, Spain, and the US have seen a clear downward trend in emissions.

In the US, CO_2_ emissions from the food production system decreased by a total of more than 30 Mt during the analyzed period, mainly due to a decrease in intermediate energy consumption. Thus, there was an opposite trend to China and India. One of the reasons for this was a significant increase in the energy efficiency of food production in the US [54, 55]. In turn, India is characterized by large environmental and energy inefficiencies in food processing and its supply, where the old technology prevails [56]. However, the main reason of increasing GHG emissions in India and China is the growth of production which requires more inputs. According to the detailed analysis of the input-output tables, in Italy and Spain, the decrease in CO_2_ emissions should be associated with the decrease in the economic activity of the food industry as a result of the global financial crisis, which has had a significant impact on these countries since 2009. Research by Karstensen et al. [57] has suggested that the slower gross domestic product (GDP) growth in the European Union countries after the global financial crisis facilitated a sustained decrease in CO_2_ emissions. On the one hand, the CO_2_ emissions from the food industry in Italy and Spain have decreased; on the other hand, the intermediate consumption of the food industry has decreased, which has had an impact on the reduction of CO_2_ emissions from the entire food production system. At the same time, CO_2_ emissions from agriculture in Spain increased during the investigated period and they decreased in Italy. As shown by Popp et al. [58], based on three different emission scenarios, the most effective way of mitigating the emissions from food production, would be the change to less animal dependent consumption patterns, as the technological improvements have their limitations. As shown by the FAO data, there was a decline in animal production in the analyzed period, especially in the years after the global financial crisis (when GHG emissions fell the fastest) in Italy, Spain and the United States. However, the latest data indicate an increase in the consumption of animal products in these countries, or the lack of clear changes in this regard, which suggests that in the future it will be difficult to reduce the absolute amount of GHG emissions.

As agricultural land can store a large amount of carbon, overall CO_2_ emissions from agriculture are small in comparison to CH_4_ and N_2_O emissions, which are the main sources of emissions in agriculture [59]. CH_4_ emissions are primarily responsible for enteric fermentation and manure management related to animal production. Depending on the region, large amounts of CH_4_ can also result from rice cultivation as well as natural or prescribed burning of savanna, which is also linked to the emission of significant amounts of N_2_O. Additionally, the main sources of N_2_O emissions in agriculture include the use of synthetic fertilizers, manure-related activities (manure applied to soils, left on pastures, manure management), crop residues, and, if present, cultivation of organic soils [60].

**Fig 2:**
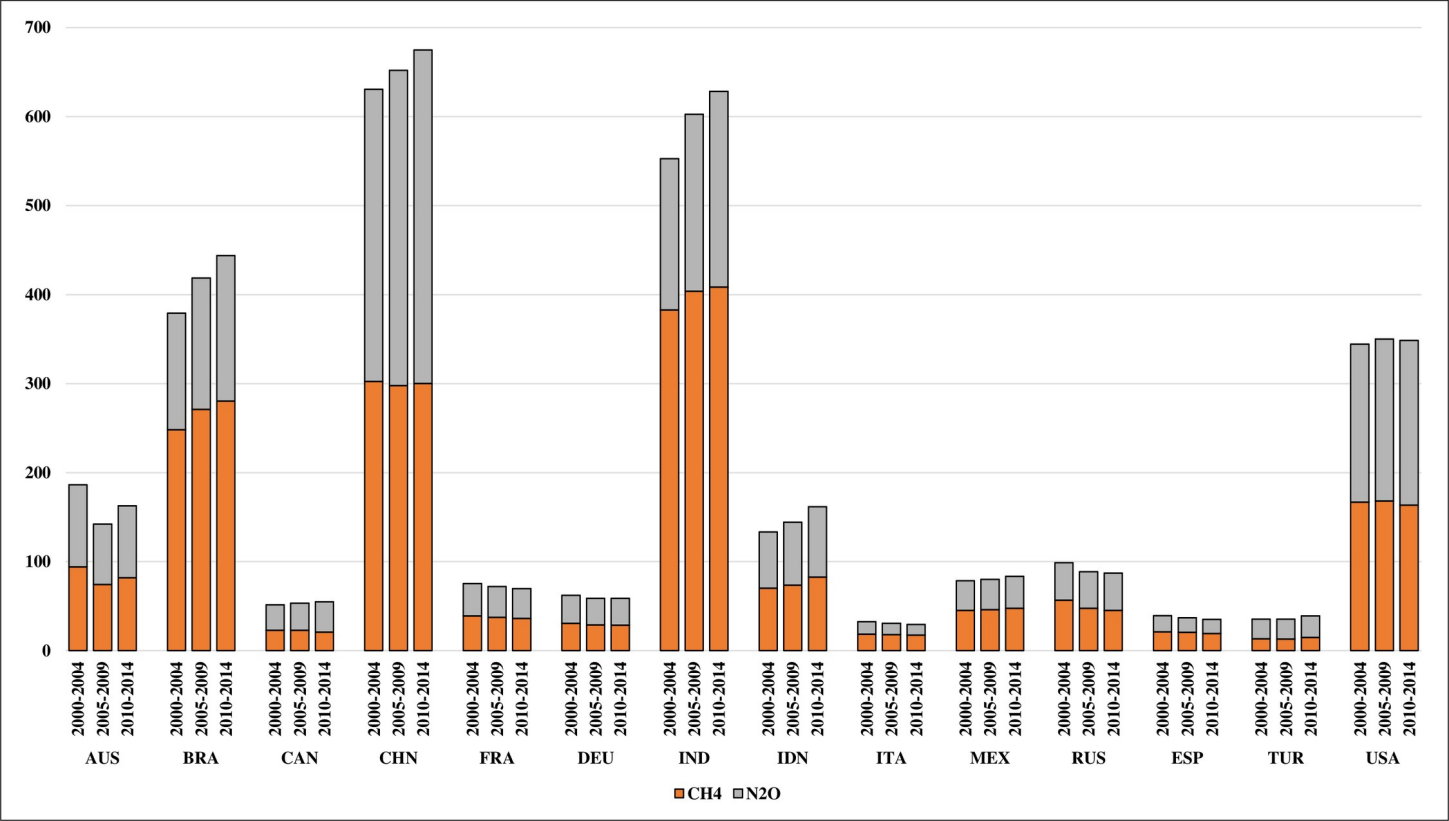
Average agricultural emissions of methane (CH_4_) and nitrous oxide (N_2_O) in the selected countries in megatonnes of carbon dioxide equivalent (CO_2_ eq). Source: Authors’ compilation based on the FAO Emissions Database, http://www.fao.org/faostat/en/#data/GT.

During the examined period, the total CH_4_ and N_2_O emissions from agriculture in the examined countries increased by more than 200 Mt CO_2_ equivalent (CO_2_eq) from about 2.7 Gt CO_2_ eq in 2000 to more than 2.9 Gt CO_2_ eq in 2014 (Fig 2). N_2_O emissions were responsible for 85% of this increase, but the share of CH_4_ emissions is still higher, although it decreased from 56% to 53% in the period under review. Usually, countries where CH_4_ emissions are predominant have a relatively high proportion of animal production; thus, they have high CH_4_ emissions due to enteric fermentation. The exception is Indonesia, where the main cereal produced is rice, whose cultivation accounts for most of the CH_4_ emissions; a relatively small amount of CH_4_ emissions comes from animal production [61].

An increase in the total CH4 and N2O emissions from agriculture occurred in most of the examined countries, i.e., Brazil, Canada, China, India, Indonesia, Mexico, Turkey, and the US. The greatest increase was recorded in India, where more than 45% of the CH4 and N2O emissions in the analyzed period came from enteric fermentation, which was also responsible for a large part of the observed increase. One of the reasons for such high emissions is the culture-based ban on the slaughter of cattle in most Indian states, which leads to maintaining a high number of animals [62]. India is also a world leader in milk production, due to its large dairy cow herds, which has a significant impact on high CH_4_ emissions [63]. However, the largest increase in emissions during the period under review was related to the increase in the use of synthetic fertilizers, which accounted for less than 13% of the total CH_4_ and N_2_O emissions in India in 2000, and more than 17% of the total emissions in 2014.

As the detailed FAO data indicate, the increased use of synthetic fertilizers was also the main reason for the increase in N_2_O emissions in China and Brazil. Furthermore, in China, a large percentage of CH_4_ emissions stems from rice cultivation. In Brazil, the main increase in CH4 and N2O emissions during that period was related to animal production, namely enteric fermentation and manure left on pastures. In Indonesia, the increase in CH4 and N2O emissions was due to the cultivation of rice as well as the increased use of synthetic fertilizers and the cultivation of organic soils. In Canada and the US, CH4 and N2O emissions also increased mainly due to increased use of synthetic fertilizers. Of the countries that increased CH_4_ or N_2_O emissions, only Turkey did not experience a significant increase in fertilizer use, and Mexico even saw its use decrease. In Turkey and Mexico, most of the increase in emissions came from manure left on pastures and enteric fermentation. These two countries have a higher share of emissions from manure left on pastures than other countries due to the specific structure of livestock, where there are relatively high shares of poultry and, in the case of Turkey, also goats and sheep.

The observed decreases in the emissions of the discussed GHGs in European countries (France, Germany, Italy, Russia, Spain) are mainly caused by the decrease in CH_4_ emissions from enteric fermentation. FAO detailed data indicate a decrease in the livestock population in these countries, especially cattle, which accounts for the largest share of CH_4_ emissions. In European countries, the evolution of food consumption patterns towards a diet less dependent on meat could be one of the reasons for these changes [64]. Moreover, France and Italy also recorded a decrease in N_2_O emissions from the use of synthetic fertilizers. In Germany and Spain, the levels of CH4 and N2O emissions remained similar during the studied period; they only significantly increased in Russia.

A specific situation related to GHG emissions has occurred in Australia, where savanna burning is the cause of a significant portion of the CH_4_ and N_2_O emissions. In the Australian savanna, fires naturally occur during the late dry season, releasing substantial amounts of GHGs into the atmosphere. To some extent, the frequency of savanna fires is random, so the resulting emissions vary annually. Controlled savanna burning in the early dry season has been introduced to limit the effects of subsequent, uncontrolled natural biomass fires, one of the aims of which is to reduce GHG emissions [65]. Consequently, the total amount of CH_4_ and N_2_O emissions in Australia fluctuates. For example, in the period 2000–2004, savanna burning accounted for over 43% of the total CH_4_ and N_2_O emissions; in the period 2005–2009, it was less than 32% and in the period 2010–2014, it was about 40%. Moreover, in Australia, in the period 2010–2014, N_2_O emissions from the use of synthetic fertilizers increased, while emissions from manure left on pastures decreased.

An important aspect of GHG emissions changes in the food production system is also the impact of climate change. The observed climate changes primarily affect the yield and differ depending on the production direction and the region of the world [66]. As indicated by Ray et al. [67] who studied the impact of climate change on the production of ten of the world’s most popular crops, it varies from -13.4% to 3.5% depending on the crop. The study also shows that climate change has a negative effect primarily in Europe, Africa and Australia, positive in South America and mixed for North America and Asia. The pursuit of high yields increases GHG emissions, and climate change may affect the size of yields to a varying degree, including determining the future directions of production.

**Fig 3:**
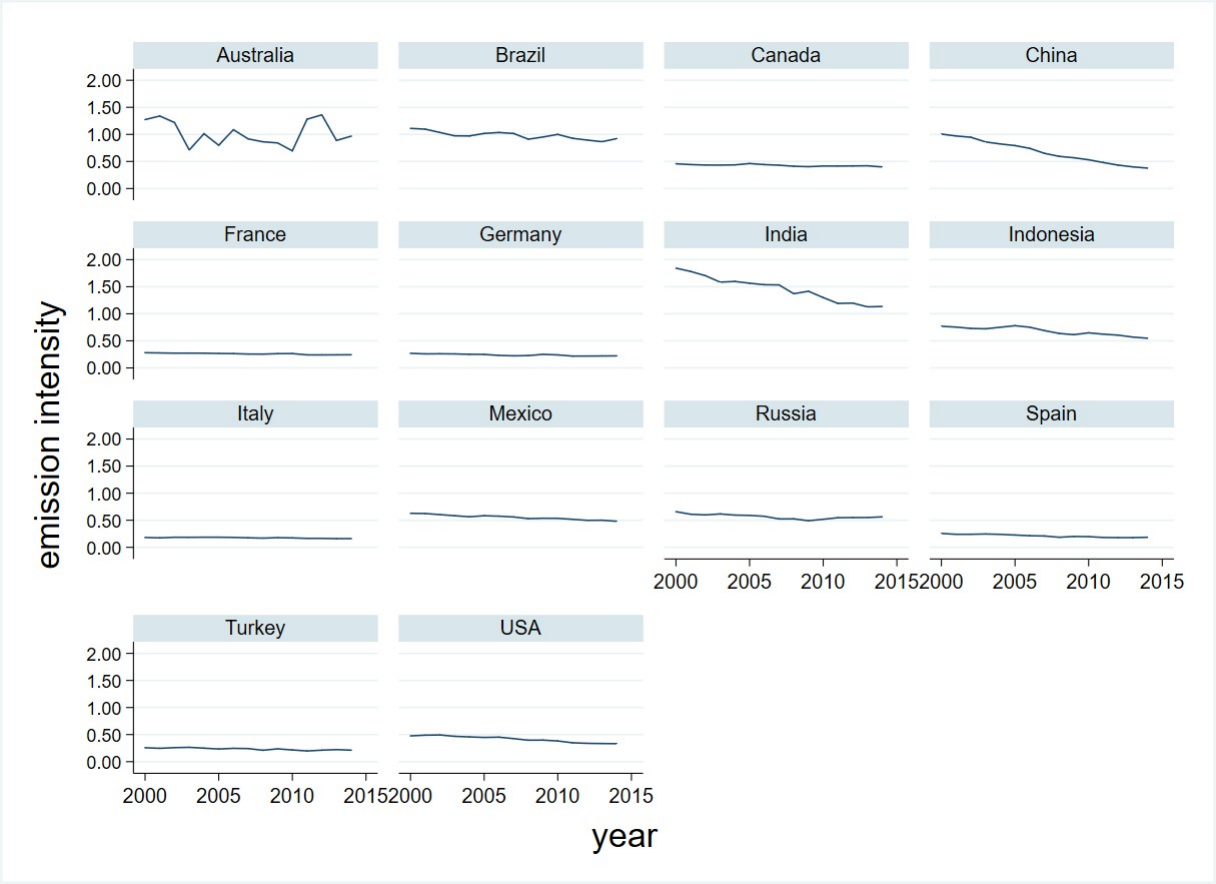
Emission intensity of food production in the examined countries in 2000–2014 (kg CO_2_ eq per USD 1 worth of food production global output). Source: Authors’ calculations (in STATA 15) based on data from WIOD Environmental Accounts (Update 2000–2016), WIOD, Release 2016 and FAO Emissions Database.

Throughout the studied period there was an increase in the investigated GHG emissions from over 3.7 Gt CO_2_ eq in 2000 to almost 4.2 Gt CO_2_ eq in 2014; however, this increase did not translate into an increase in emission intensity, which decreased from over 0.68 kg CO_2_ eq per USD 1 worth of food production global output in 2000 to less than 0.46 in 2014. This shows that, on average, the increase in the value of food production was significantly faster than the GHG emissions it causes. In all the studied countries, excluding Australia, a downward trend in emission intensity was observed in the investigated period (Fig 3). It was particularly significant in China and India. In China, the index fell from over 1 kg CO_2_ eq per USD 1 worth of food production global output in 2000 to 0.38 in 2014. In India, it fell from 1.84 kg CO_2_ eq per USD 1 worth of food production global output in 2000 to 1.13 in 2014. The index figures in Australia were influenced by the previously mentioned annual fluctuations in GHG emissions.

**Fig 4:**
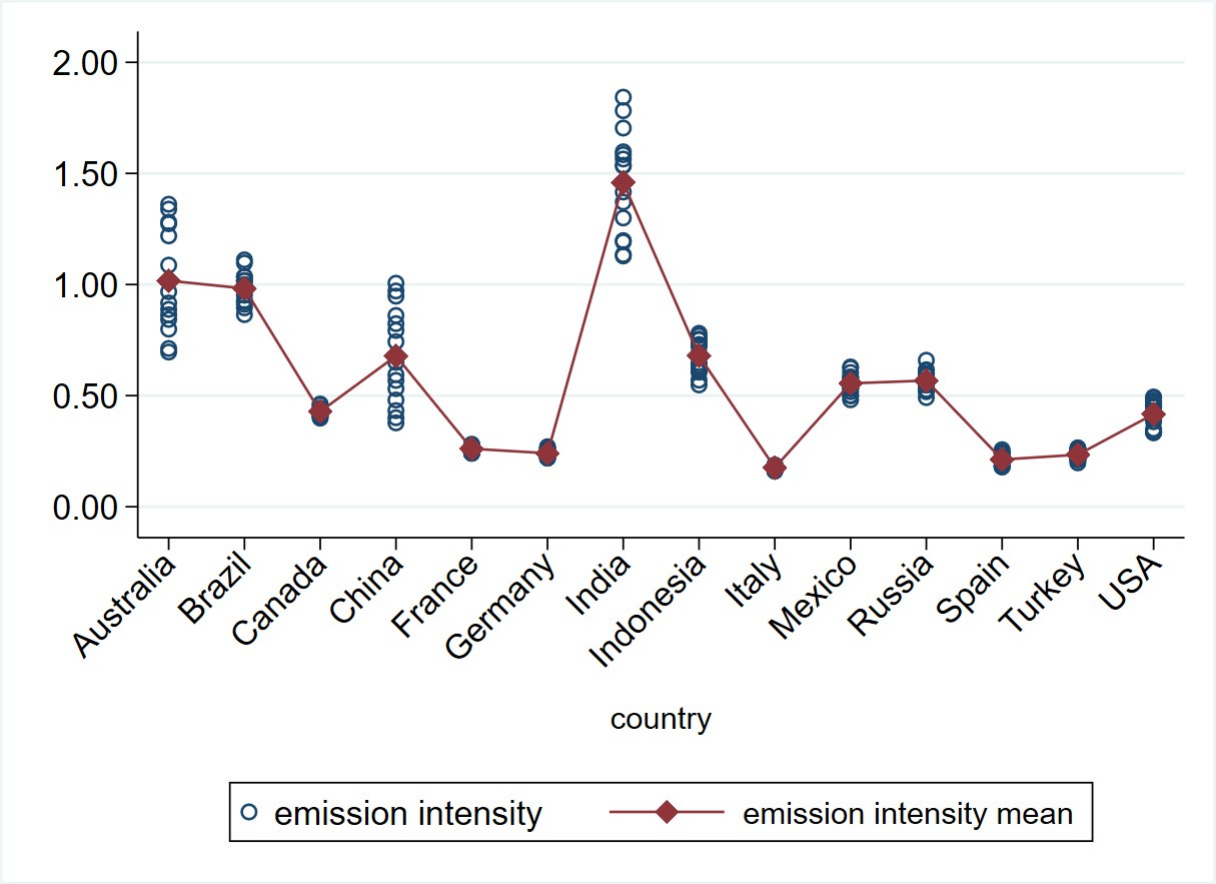
Heterogeneity of emission intensity across countries. Source: Authors’ calculations (in STATA 15) based on data from WIOD Environmental Accounts (Update 2000–2016), WIOD, Release 2016 and FAO Emissions Database.

**Fig 5:**
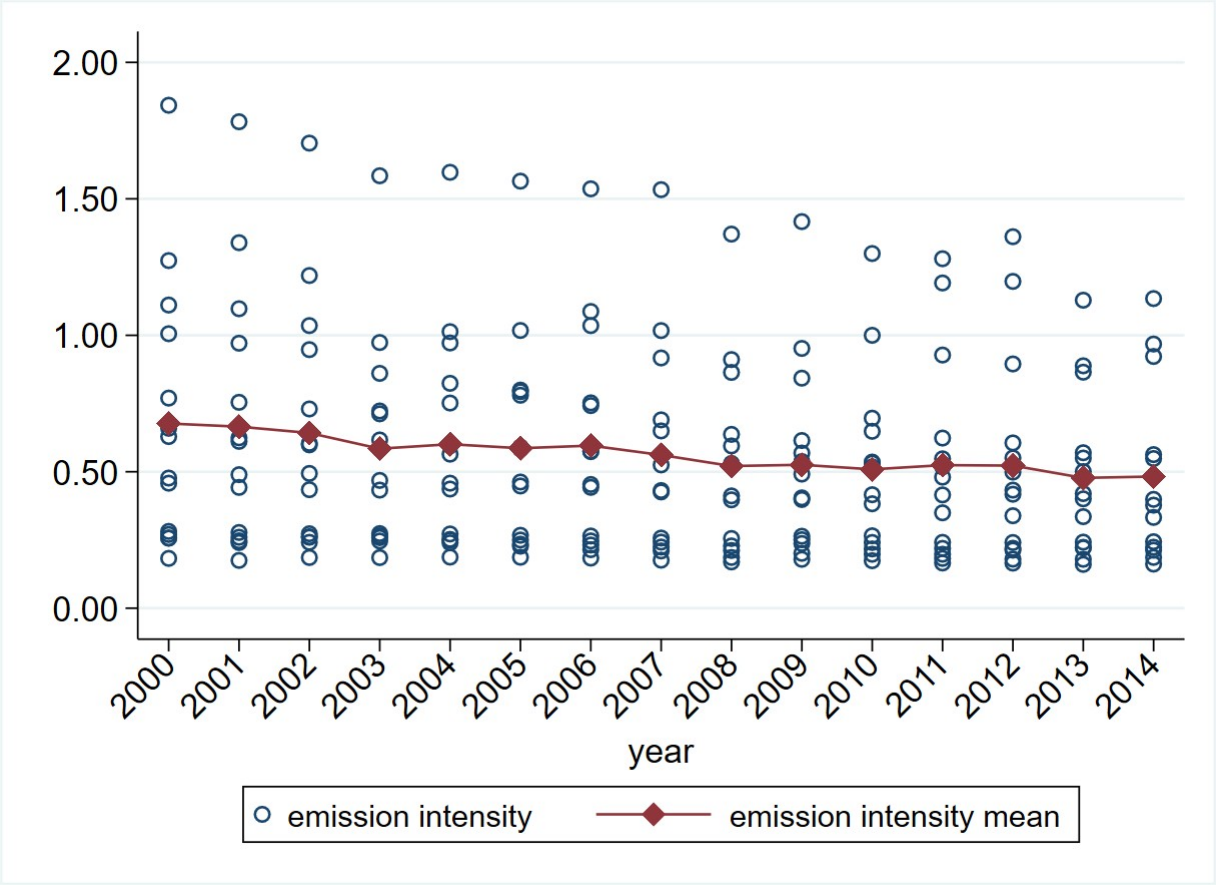
Heterogeneity of emission intensity across years. Source: Authors’ calculations (in STATA 15) based on data from WIOD Environmental Accounts (Update 2000–2016), WIOD, Release 2016 and FAO Emissions Database.

Australia, China, and India demonstrated the highest variability of emission intensity during the analyzed period; the lowest variability was observed in Italy and France (Fig 4). From year to year, the absolute difference in the emission intensity values between the examined countries decreased (Fig 5); on average, India, Australia, and Brazil had the highest values, while Italy, Spain, and Turkey had the lowest. Thus, the value of the variable changes over the years, but it also changes from country to country. In this situation, it seems advisable to conduct research based on panel data, and not just cross-sectional data or time-series data.

### Determinants of emission intensity

The determinants of changes in the value of the emission intensity of the food production system in the studied countries are crucial from the point of view of the aim of the study. The results of the panel regression model on these determinants are presented in Table 2.

**Table 2:** Emission intensity of food production systems. Linear regression, correlated panels corrected standard errors (PCSEs).

emission_intensity	Coef.	Std. Err.	z	P>|z|	[95% Conf. Interval]	
GDP_per_capita	6.91e-06	2.92e-06	2.37	0.018	1.18e-06	.0000126
HDI	-2.676769	.2911239	-9.19	0.000	-3.247362	-2.106177
nitrogen_fertilizers_use	-.0076713	.0007444	-10.31	0.000	-.0091303	-.0062123
nitrogen_fertilizers_production	.0032048	.000673	4.76	0.000	.0018858	.0045237
agriculture_material_intensity	-.0045798	.0014855	-3.08	0.002	-.0074915	-.0016682
food_industry_material_intensity	-.005046	.0029009	-1.74	0.082	-.0107316	.0006396
energy_use_per_capita	.0000393	.0000143	2.74	0.006	.0000112	.0000674
fossil_fuel_energy_use	-.0140933	.0008417	-16.74	0.000	-.015743	-.0124436
population_density	.002482	.0002029	12.23	0.000	.0020843	.0028796
agricultural_area	6.52e-07	1.58e-07	4.12	0.000	3.42e-07	9.62e-07
agricultural_area_per_person	.0312663	.0049964	6.26	0.000	.0214736	.041059
yield_of_cereals	-.0036678	.0017289	-2.12	0.034	-.0070565	-.0002792
animal_production	.0094037	.0014188	6.63	0.000	.0066228	.0121845
_cons	3.550733	.2334062	15.21	0.000	3.093265	4.008201

Source: Authors’ calculations (in STATA 15) based on data from WIOD Environmental Accounts (Update 2000–2016), WIOD, Release 2016 and FAO Emissions Database.

The research findings show that the increase in GDP per capita results in an increase in emission intensity. With respect to existing studies on GDP per capita and environmental change, this result is not apparent. For example, Ozcan [68] showed that GHG emissions change with economic progress. For example, energy consumption is expected to increase more slowly than GDP growth in the post-industrial stage; thus, pollutant emissions per unit of production will decrease [69]. The positive relationship between GDP per capita and emission intensity could have been determined by the global financial crisis of 2007–2009 [70]. It can be seen that, in most of the investigated countries, the emission of pollutants per unit of production showed a downward trend, while the value of GDP per capita had a general upward trend, except for the years in which the GDP per capita dynamics slowed due to the financial crisis. This is due to the fact that, in some countries, lower values of emission intensity of the food production system were obtained under conditions of lower GDP per capita. Thus, in countries with a higher GDP per capita, the emission intensity of the food production system was often higher.

The impact of the financial crisis on the relationship between GDP per capita and emission intensity is confirmed by the Human Development Index (HDI) results. HDI is a more qualitative index of development than GDP per capita, and only one of its components is per capita income, which makes its values less vulnerable to economic crises. The regression results indicate that an increase in HDI values leads to a decrease in the emission intensity of food production systems. Overall, in the countries with higher HDI, GHG emissions per unit of production are decreasing. This is due to a higher level of education of the population, which increases environmental awareness, and the demand for luxury goods, which also includes good quality of the natural environment [71]. In countries with a high HDI, the importance of modern, eco-efficient technologies is also increasing, which determines changes in food production, among others.

The regression results indicate that the use of nitrogen fertilizers causes a decrease in emission intensity. This has occurred despite the fact that the use of fertilizers increases GHG emissions, which indicates their significant positive impact on production volumes. Therefore, the use of nitrogen fertilizers increases the value of global food production faster than it increases GHG emissions. This situation is consistent with the results of N_2_O emissions, where synthetic fertilizers are responsible for the highest increase. However, at the same time, the overall level of emission intensity in the examined countries was also decreasing. As the research results show, unlike in the case of the use of nitrogen fertilizers, an increase in their production contributes to an increase in the emission intensity of food production systems. First, this is due to the low correlation between the production and use of nitrogen fertilizers. According to the detailed FAO data, throughout the entire considered period, only Russia, Canada, and Indonesia were self-sufficient in terms of nitrogen fertilizer production, and in most of those years, China was, too. The rest of the countries were strongly dependent on imports; hence, the difference between the impact of the direction of fertilizer production and fertilizer use on emission intensity. The production of nitrogen fertilizers increases GHG emissions because, to a large extent, its production process requires emission-intensive fossil fuels [72].

The material intensity of agriculture is another variable, the increase of which causes a decrease in the emission intensity of food production systems. A higher share of intermediate consumption in global output increases GHG emissions, mainly due to the fact that fertilizers and pesticides or the fuel and energy industry usually have a high share of intermediate consumption of agriculture [73]. However, as Baer-Nawrocka and Mrówczyńska-Kamińska [74] indicated, apart from the differences resulting from the directions of production, in countries with higher production value, the material intensity of agriculture is significantly higher. This is due to the high labor intensity of agriculture in developing countries, which also have a lower value of output. Due to the release of labor to other sectors of the economy, labor is substituted by capital in agriculture. This leads to a situation in which, after this process is concluded, an increase in intermediate consumption is necessary to increase output [45]. Intuitively, a similar situation should be expected in the case of the relationship between the material intensity of the food industry and the emission intensity of food production systems; however, in this case, the obtained results were not statistically significant.

Energy consumption per capita is another variable that was examined. It is a determinant of the increase in the emission intensity of food production systems. It has been reported that, at least until 2050, global energy production will depend on fossil fuels [75], which account for a large proportion of GHG emissions. To minimize GHG emissions, incentives for the use of renewable energy sources in the future have been introduced; this will increase the share of clean energy in total energy use [76]. Currently, however, higher energy consumption per capita generally means higher GHG emissions, which increase the emission intensity of food production systems.

The share of the use of energy from fossil fuels in the total energy consumed in the economy is another variable that was examined. Paradoxically, an increase in the value of this variable causes a decrease in the emission intensity of food production systems. The use of fossil fuel energy increases the amount of GHG emissions in the atmosphere. However, an increase in the consumption of energy from conventional sources is closely related to mechanization or technical development, which positively affect the volume of food production. A higher share of fossil fuels in total energy use is a determinant of the decrease in emission intensity of food production systems because, as currently used, food production techniques are almost entirely dependent on fossil fuels [77, 78], the use of which significantly increases global output.

According to the calculations, the increase in population density is one of the determinants of the increase in emission intensity of food production systems. The world’s population is growing, and each additional person produces waste or consumes energy, resulting in higher GHG emissions. In the case of higher population density in a given country, this leads to higher emissions per area unit. These conclusions can be drawn, for example, from the study by Liu et al. [79], which observed the relationship between population density and GHG emissions by examining population migration in China.

An increase in the utilized agricultural area measured as an absolute number of hectares of agricultural area, or per capita, also increases the emission intensity. Thus, the more area a country has to produce food, the less effective it is in the sense of environmental stewardship. Although with more intensive production, GHG emissions are increasing in absolute terms, this increase is less dynamic than the increase in food production. This is also confirmed by the negative relationship between emission intensity and yields: the higher the yield, the lower the GHG emission per production unit. Although this assumption is theoretical, a more detailed analysis may confirm this relationship. Perhaps this is because more developed countries, where production intensity is higher, produce food more sustainably. In food production, it is essential that the consumption of the means of production is proportionate. One-sided use of one of the elements influencing productivity can only have a relatively small impact on the yield. This can be linked to Liebig’s law that the scarcest resource (in relation to needs) has a limiting effect on production [80]. This law can also be applied to agricultural production. For example, high fertilizer consumption without protection from agrophages will result in significant GHG emissions (in terms of fertilizer consumption and production) that will not be accompanied by simultaneous dynamic yield increases [81]. In countries with highly developed agriculture, some practices exist that increase yields and also have a positive impact on emissions. These practices may include the use of intercrop or modern, simplified cultivation technologies. Modern technologies are also used in these countries, which significantly improves the precision of the industrial means of production. All these factors make the emission intensity decrease, despite higher yields.

The share of animal production in total agricultural production is another factor that influences the increase in the emission intensity of food production. This conclusion is not surprising because several studies have indicated that meat-based food production has a much lower environmental performance. The production of both energy and protein through animal production involves several-times more GHG emissions than other types of production. For example, Poore and Nemecek [7] showed that the production and consumption of meat contributes creates nearly four-times more GHG emissions than a plant-based diet.

## Conclusions

This study on the emission intensity of food production systems for countries that are responsible for more than 65% of global food production showed that between 2000 and 2014 there was an average increase in GHG emissions; however, that increase was slower than the observed increase in global output. This translated into an overall decrease in emission intensity from over 0.68 kg CO_2_ eq per USD 1 worth of food production global output in 2000 to less than 0.46 in 2014. Few previous studies have examined GHG emissions in terms of production value. Additionally, the study’s main contribution to the existing literature is its analysis of the determinants of the emission intensity of food production systems carried out based on a panel regression model. The results indicate that some of the variables, which are associated with the impact on the increase in GHG emissions, are determinants of the decrease in emission intensity. In particular, the use of nitrogen fertilizers or the material intensity of agriculture indicates that these variables have a stronger impact on the growth of global output than on the growth of GHG emissions in food production systems. The results are significant from the point of view of the projected growth of the world’s population.

The study is based on the assumption that the food production system consists of agriculture, the food industry, and all the components of other sectors that provide inputs to agriculture and the food industry. In this way, the emphasis is placed on the food production process itself, excluding, for example, the transport of the produced food, which is responsible for a significant percentage of the GHG emissions in the food chain. This study has some limitations. First, only CO_2_, CH_4_, and N_2_O emissions were taken into account. In fact, the total amount of emissions is also made up of other GHGs, but their importance in the food production system is minimal. The second limitation is the level of data aggregation; part of the agricultural production that is non-food production was also taken into account. However, according to FAO data, the percentage of that production was less than 2.5% on average in the countries that were analyzed in this study.
